# Supplementary material for: Structure of the human ATAD2 AAA+ histone chaperone reveals mechanism of regulation and inter-subunit communication
Source: Commun Biol. 2023 Sep 28;6:993. doi: 10.1038/s42003-023-05373-1 (PMC10539301; doi:10.1038/s42003-023-05373-1)
Supplement: Supplementary file 2 — Supplementary Information [file 42003_2023_5373_MOESM2_ESM.pdf]

Supplementary Information for

Structure of the Human ATAD2 AAA+ Histone Chaperone Reveals Mechanism of Regulation and Inter-subunit Communication

Carol Cho, Christian Ganser, Takayuki Uchihashi, Koichi Kato, and Ji-Joon Song

Correspondence to: carol.cho@kaist.ac.kr and songj@kaist.ac.kr

SUPPLEMENTARY FIG 1

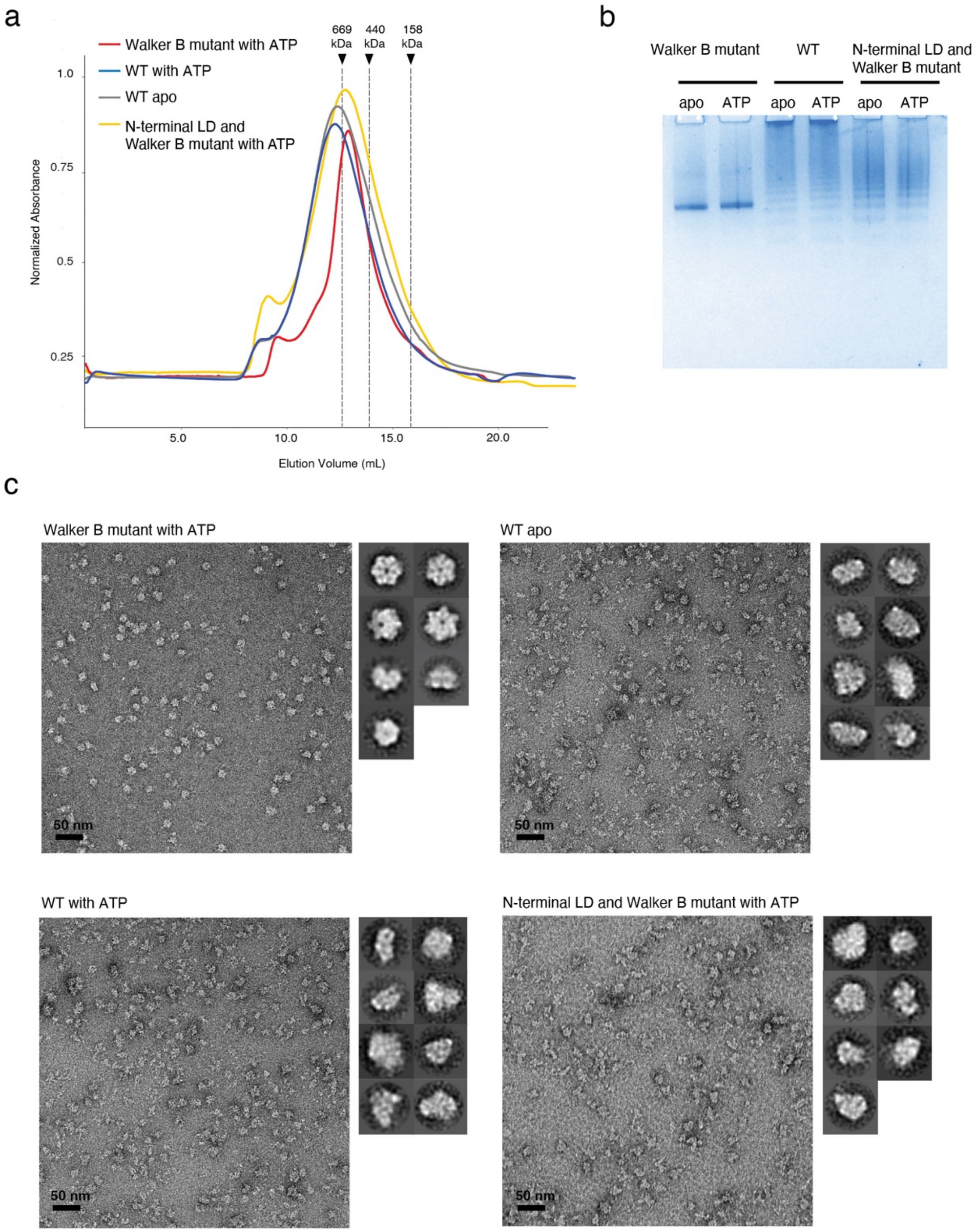

Supplementary fig 1. Oligomeric assembly of ATAD2

a Gel filtration traces of N-terminally truncated (aa 403-1390) WT and mutant ATAD2 proteins

(Walker B mutant is E532Q, while N-terminal LD and Walker B mutant is

D415A/E532Q/R540A) run over a Superose 6 increase 10/300 GL column. Peak elution volumes of gel filtration molecular weight markers (thyroglobulin (669 kDa), ferritin (440 kDa), and aldolase (148 kDa) are indicated with arrowheads. The expected molecular weight for N-terminally truncated WT or mutant ATAD2 proteins is 696kDa in the hexameric state, and 116kDa in the monomeric state. **b** Native PAGE analysis of purified WT and mutant ATAD2 proteins showing the homogeneity of ATAD2 Walker B mutant compared to WT and N-terminal LD mutant ATAD2. **c** Negative stain electron micrographs (38,000x magnification) of gel filtration peak fractions of WT and mutant ATAD2 proteins. Representative 2D class averages obtained from ~2,000 particles are shown at right of each micrograph.

SUPPLEMENTARY FIG 2

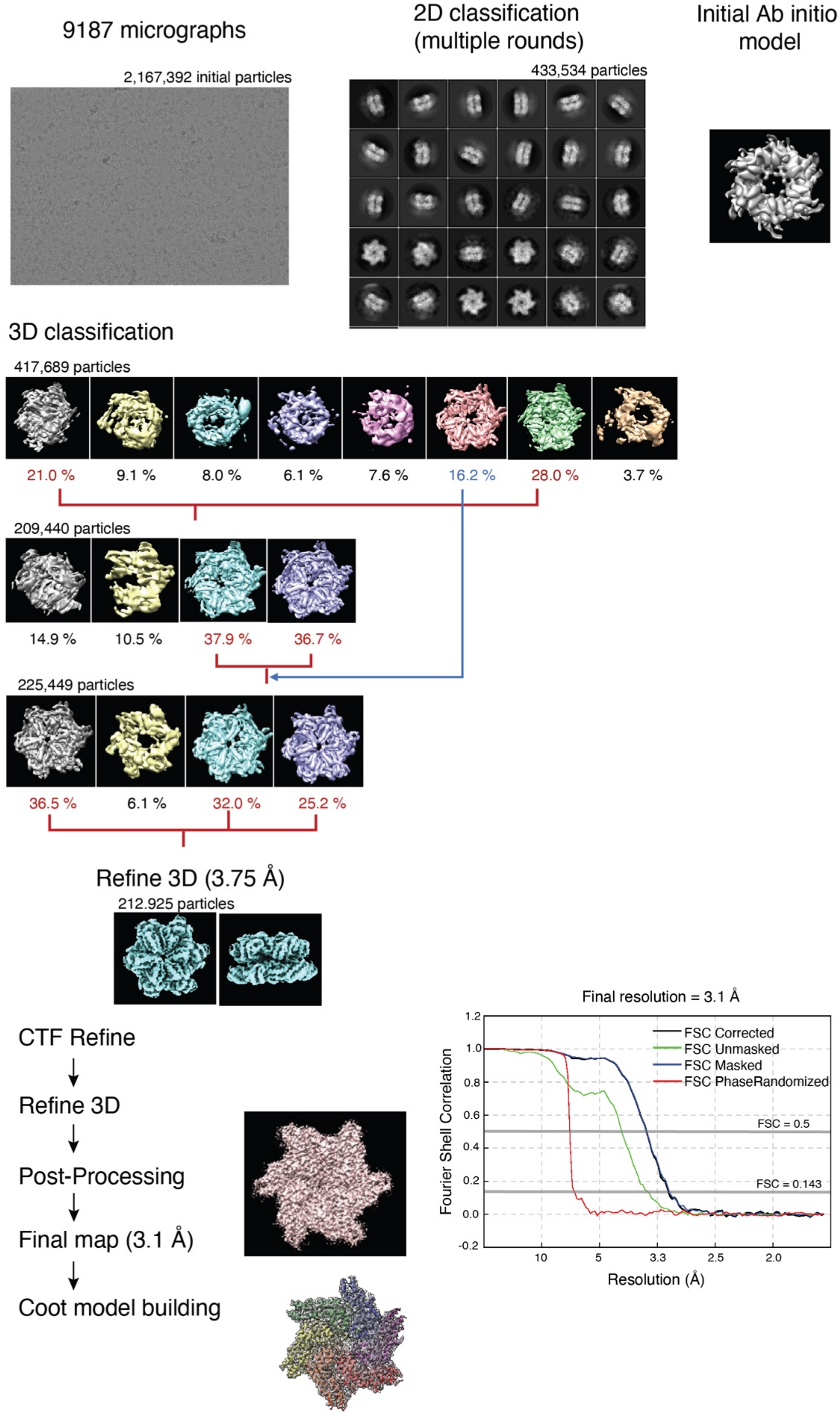

Supplementary fig 2. Workflow of cryo-EM data processing of ATAD2 Walker B mutant

Cryo-EM data processing workflow of ATAD2 Walker B mutant with ATP by Relion 3.1 and the Fourier Shell Correlation (FSC) curve of the homogenous cryo-EM refinement in Relion 3.1.

SUPPLEMENTARY FIG 3

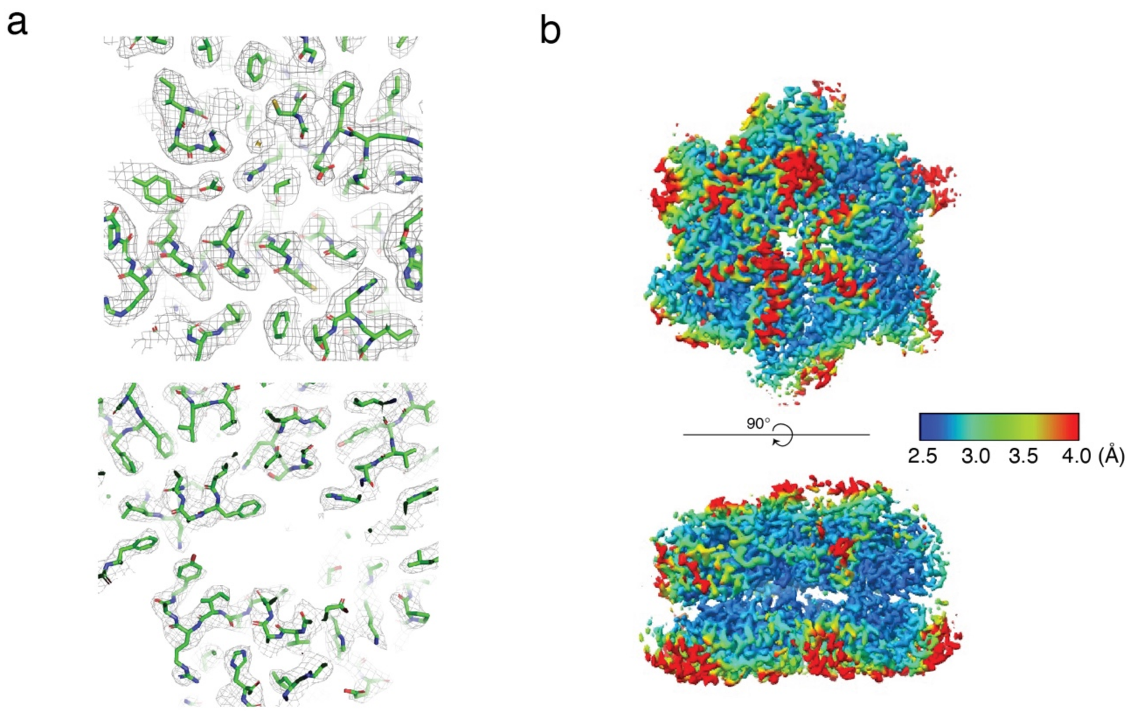

Supplementary fig 3. Local resolution and model fit of ATAD2 Walker B mutant electron density map

a Representative electron density maps of ATAD2 contoured at σ=3.0 with model of ATAD2 showing slices of AAA1 nucleotide binding domain (top) and AAA2 and helical bundle domain (bottom). **b** Top (top) and side (bottom) views of ATAD2 Walker B mutant cryo-EM maps colored by local resolution. Local resolution map was calculated by final half maps in cryoSPARC.

SUPPLEMENTARY FIG 4

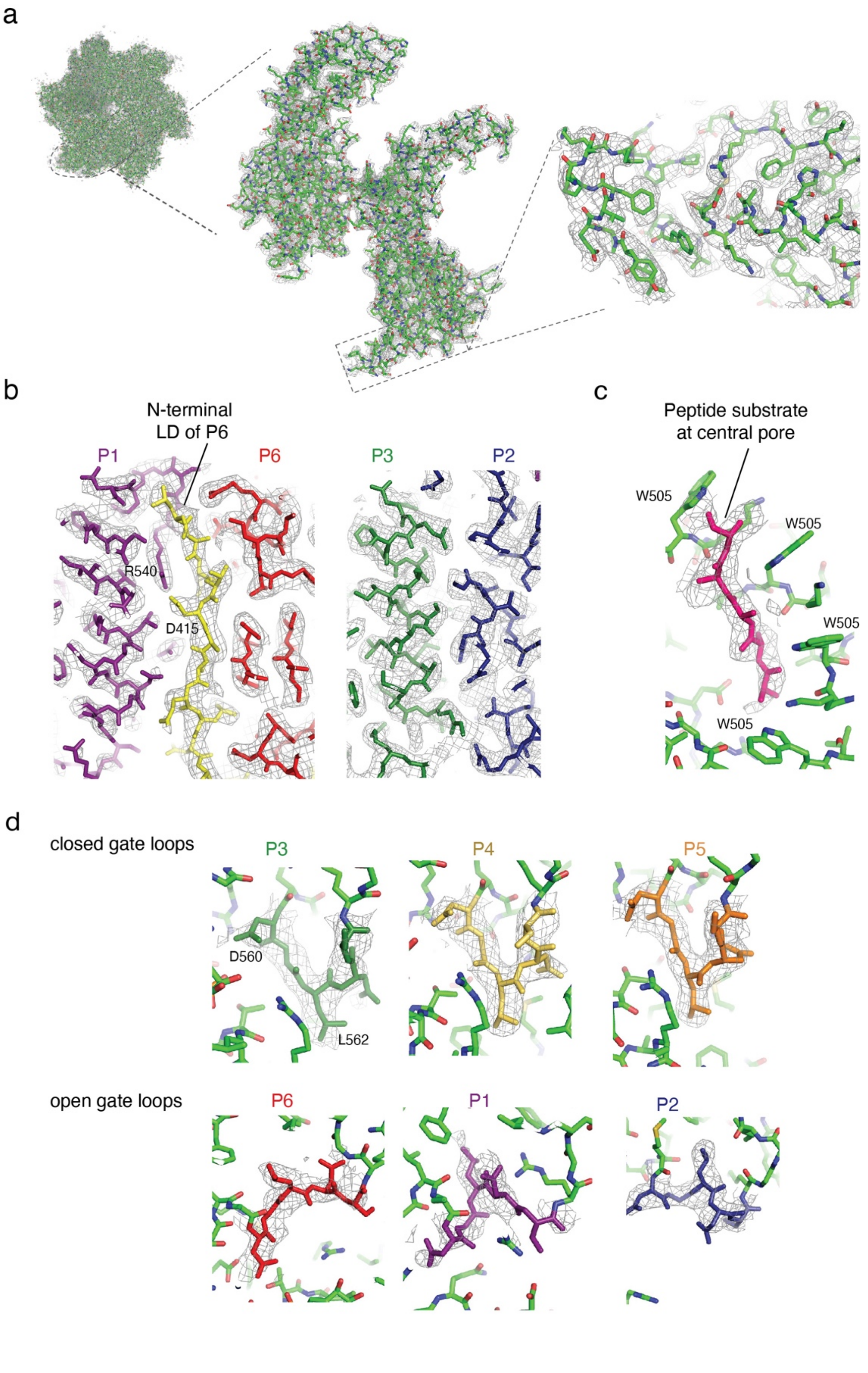

Supplementary fig 4. Detailed density maps of key structural elements in the ATAD2 Walker B mutant

a Overview of electron density map and model fit of ATAD2 Walker B mutant hexamer (left), a single chain (subunit P2) of the ATAD2 Walker B mutant hexamer (middle), and a zoomed in region of subunit P2 AAA1 helical bundle domain (right). **b** Electron density map and model fit of ATAD2 Walker B mutant P1/P6 interface and P3/P2 interface. N-terminal LD is unique to the P1/P6 interface, while all other interfaces are similar to the P3/P2 interface. **c** Electron density map and model fit of the peptide substrate (deep pink) at the AAA central pore. The peptide substrate is surrounded by a tryptophan (W505) pore loop staircase. **d** Electron density map and model fit the of gate loops grouped by conformation (top: closed gate loops, bottom: open gate loops). All density maps are contoured at σ=4.5.

SUPPLEMENTARY FIG 5

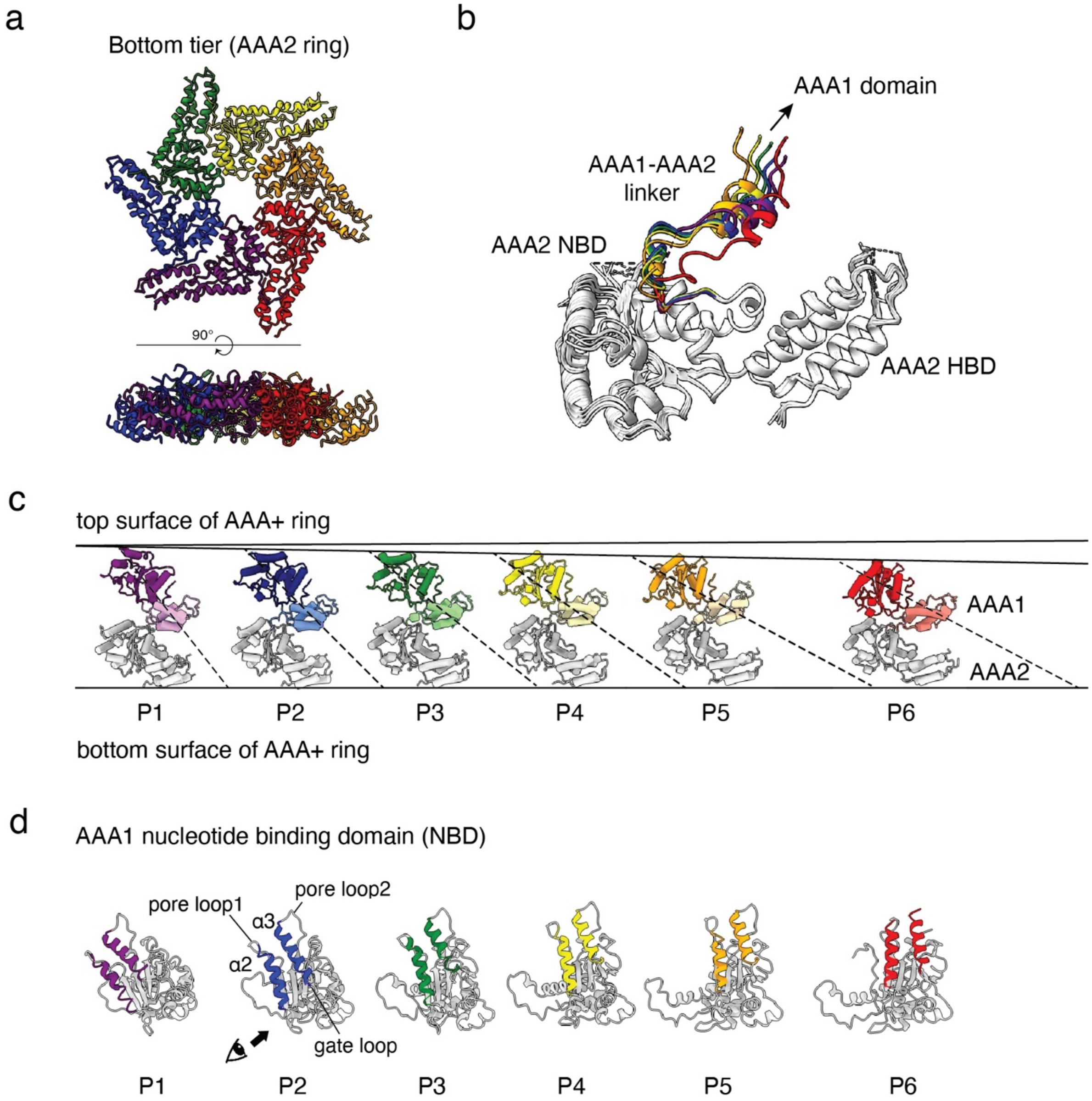

Supplementary fig 5. Structural elements contributing to ATAD2 hexamer asymmetry

a Symmetric and planar structure of AAA2/C-terminal domain ring. **b** Comparison of AAA1-AAA2 linker conformation in different ATAD2 subunits. Subunits were aligned and superimposed by the AAA2 HBD. Only AAA2 domains and AAA1-2 linkers are shown with AAA1-2 linkers colored according to subunit. **c** Comparison of subunit height and angle of AAA1 (NBD in dark hue, HBD in light hue) with respect to AAA2 (light gray) when subunits

are aligned by AAA2 HBD. **d** Comparison of AAA1 domain angle with respect to AAA2 domain in ATAD2 subunits P1-P6. AAA1 domains are aligned with respect to the AAA2 small domain (AAA2 domains not shown) and shown from a top view of the AAA ring with helix α2 and α3 colored for reference. For comparison of gate loop conformations, view from direction of arrow is shown in Fig. 3c.

SUPPLEMENTARY FIG 6

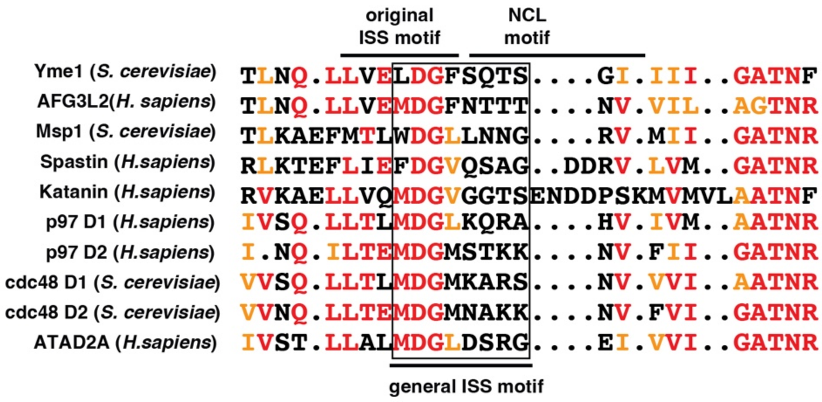

Supplementary fig 6. Sequence alignment of ɑ3-β4 loop in different AAA+ ATPases

Alignment of the ɑ3-β4 loop of various AAA+ ATPases, showing the original ISS motif as defined by the DGF tripeptide in the C-terminus of ɑ3 in m-AAA+ proteases (Yme1 and AFG3L2), the NCL motif in meiotic clade AAA+ ATPases (Msp1, spastin, and katanin), and an updated "general ISS motif" that applies to the ɑ3-β4 loop of many AAA+ ATPases such as those shown here. Residue positions that are > 70 % identical are colored red, while positions that are 70 % > similar are colored orange.

SUPPLEMENTARY FIG 7

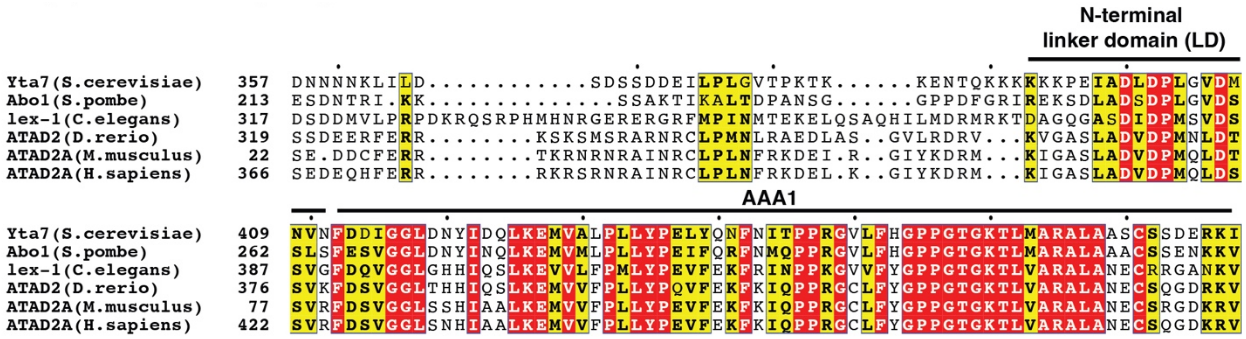

Supplementary fig 7. Conservation of ATAD2 N-terminal linker domain (LD)

Multiple sequence alignment of the N-terminal linker domain and AAA1 domains of ATAD2 homologs showing the conservation of the LD. Red represents 100 % identity while yellow represents a similarity score of > 70 %.

SUPPLEMENTARY FIG 8

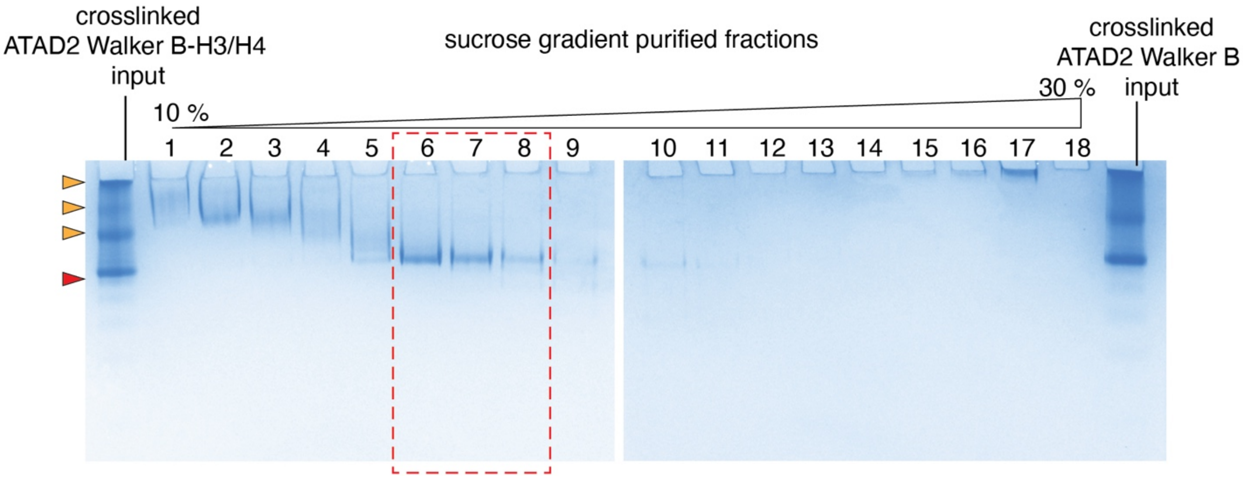

Supplementary fig 8. Chemical crosslinking and sucrose gradient purification of ATAD2-H3/H4 complex.

DSS-crosslinked ATAD2-H3/H4 complex, purified sucrose gradient fractions of the ATAD2-H3/H4 complex, and DSS-crosslinked ATAD2 run on a native Bis-tris 4-16 % gradient gel. Red arrowhead indicates bands representing a hexameric ATAD2 complex, and yellow arrowheads indicate bands with dodecameric ATAD2 or larger molecular weight complexes. Dotted red box indicates fractions used for cryo-EM or XL-MS data collection. Crosslinked ATAD2 shows similar band patterns as ATAD2-H3/H4 on a native gel.

SUPPLEMENTARY FIG 9

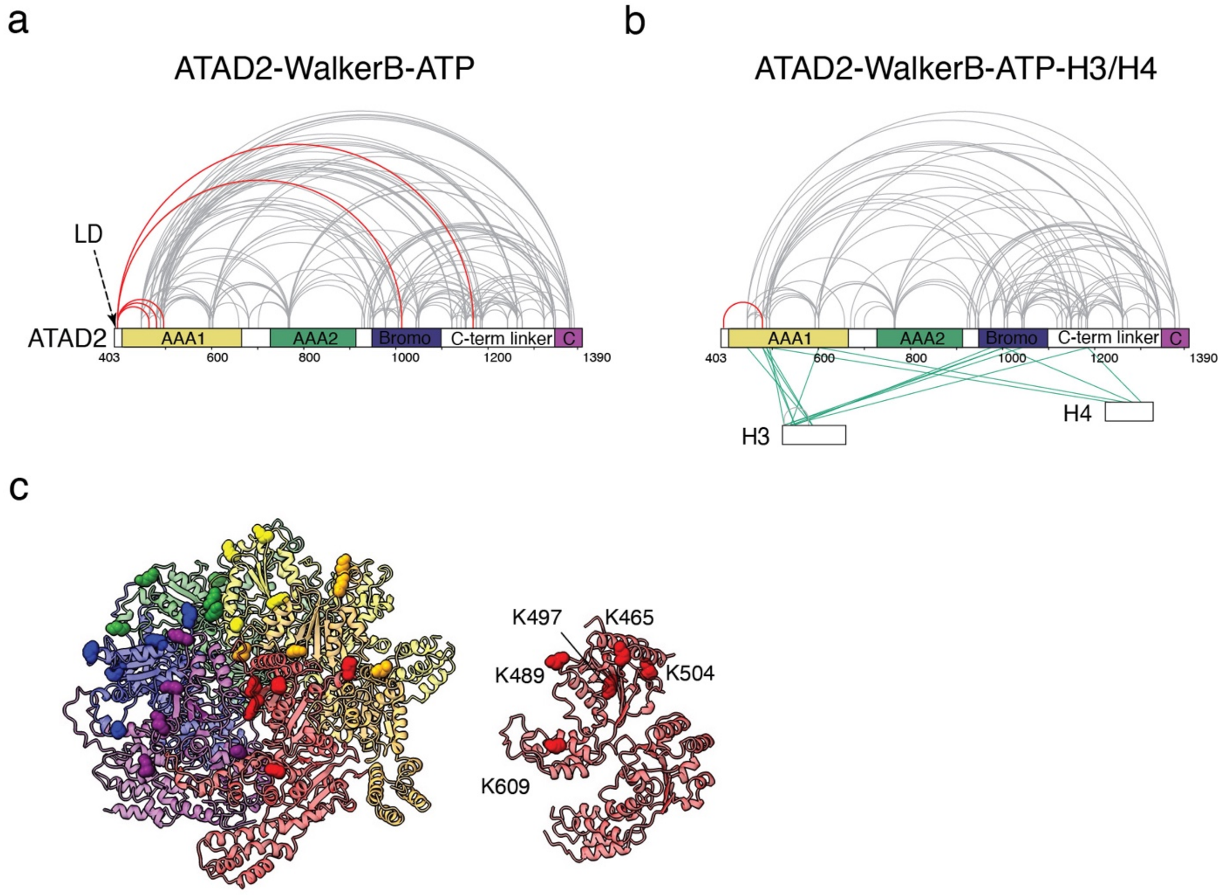

Supplementary fig 9. Crosslinking mass spectrometry (XL-MS) of ATAD2-H3/H4 complex

a Intramolecular crosslinks of ATAD2 Walker B mutant with ATP. (Crosslinks were analyzed by xQuest/xProphet and filtered by an xQuest LD score cutoff of 25 corresponding to an estimated false discovery rate of < 2 %.) Crosslinks of the N-terminal LD that disappear in the ATAD2 Walker B mutant - H3/H4 complex are colored red. **b** Intra- and inter-molecular crosslinks of ATAD2 Walker B mutant-H3/H4 complex with ATP. **c** ATAD2 residues that crosslink with histone H3/H4, mapped onto the ATAD2 hexamer (left), and monomer (right).

SUPPLEMENTARY FIG 10

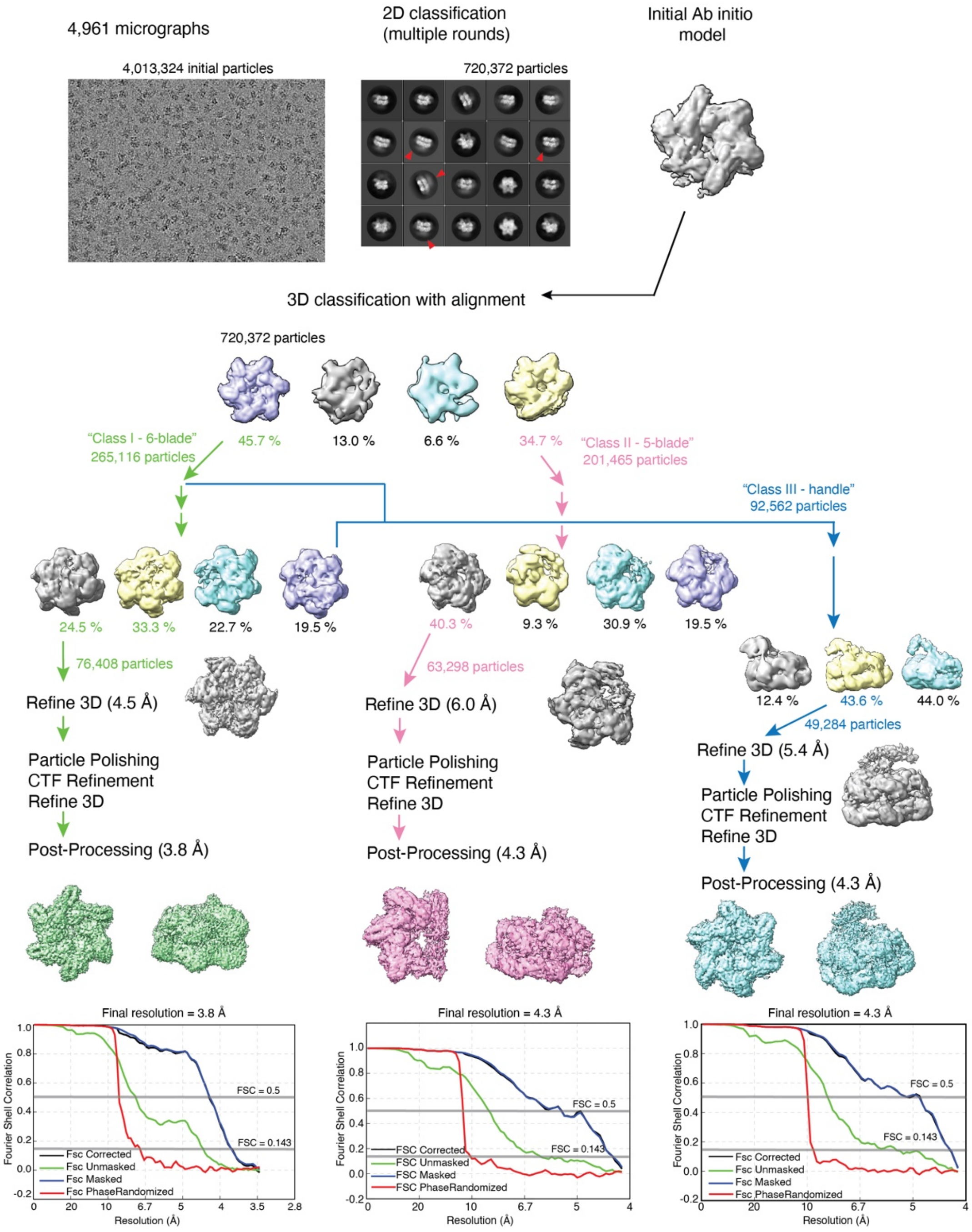

Supplementary fig 10. Workflow of cryo-EM data processing of ATAD2 Walker B mutant-H3/H4K5Q complex

Overview of cryo-EM data processing workflow of ATAD2 Walker B mutant-H3/H4K5Q complex with ATP by Relion 4.0. Red arrowheads in 2D class averages indicate structures that are similar to the “handle-like” extra density observed in Class III. 3D classification results in three major classes - Class I (6-blade), Class II (5-blade), and Class III (handle-like). Fourier Shell Correlation (FSC) curves for all classes are shown with the reported final resolution at Fsc = 0.143.

SUPPLEMENTARY FIG 11

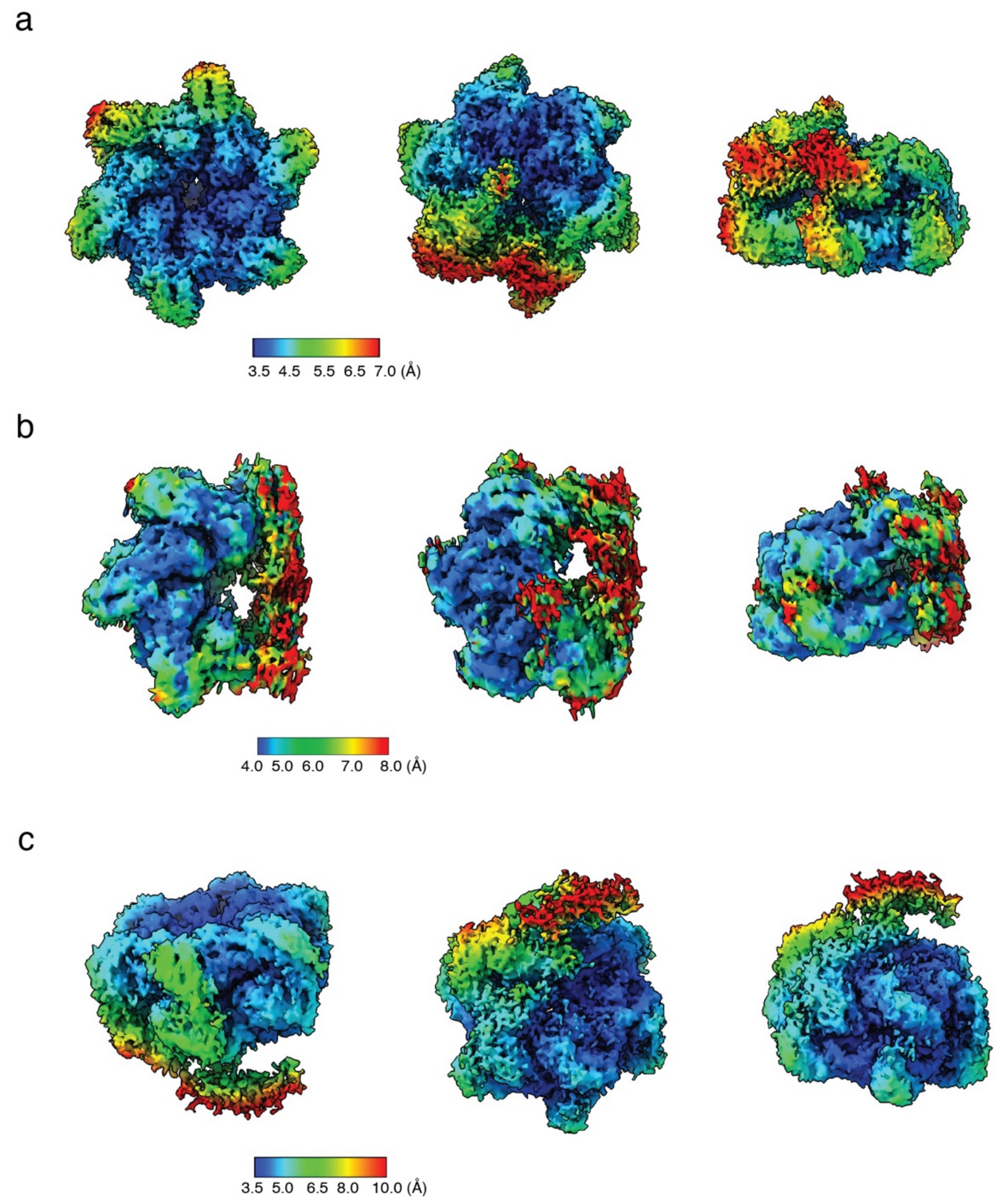

Supplementary fig 11. Local resolution maps of ATAD2 Walker B mutant-H3/H4 complex

a-c Views (bottom, top, and side) of ATAD2 Walker B mutant-H3/H4K5Q cryo-EM maps class I (**a**), class II (**b**) and class III (**c**) colored by local resolution. Local resolution maps were calculated by final half maps in ResMap.

SUPPLEMENTARY FIG 12

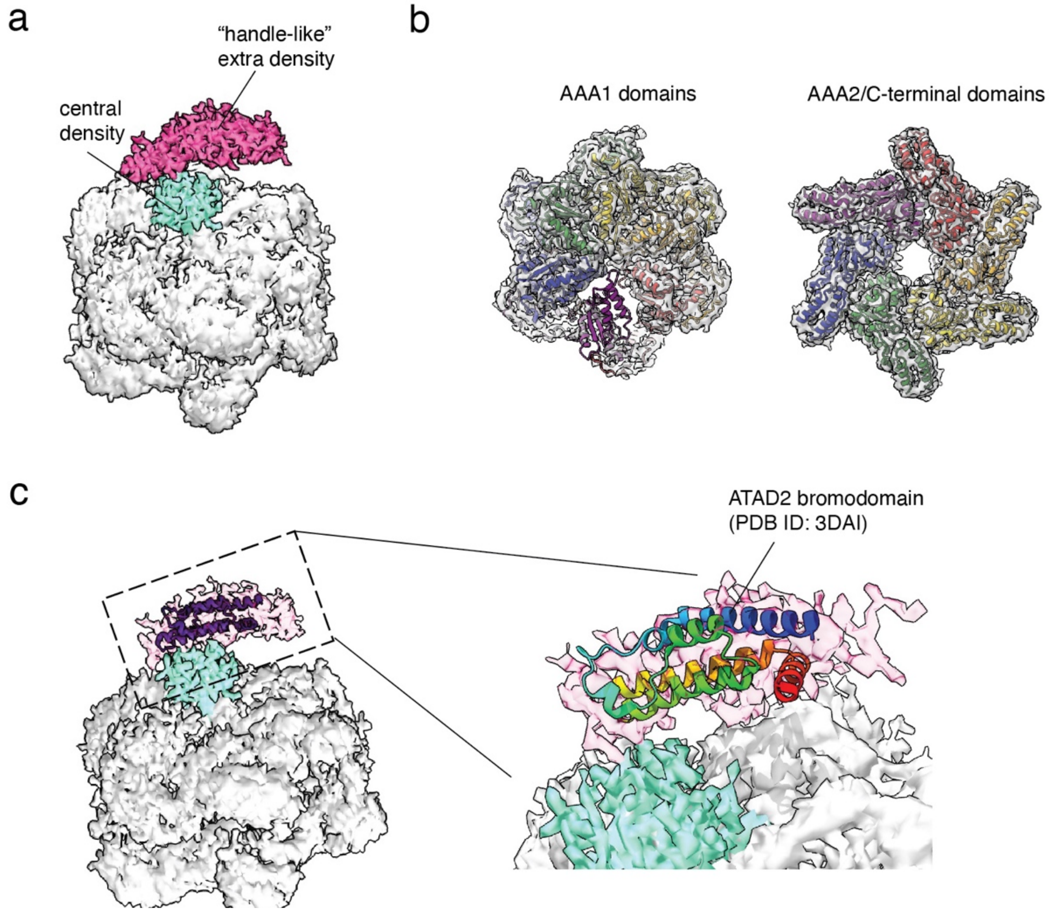

Supplementary fig 12. Cryo-EM map and model of ATAD2 Walker B mutant-H3/H4K5Q complex Class III

a Cryo-EM map of ATAD2 Walker B mutant-H3/H4K5Q complex Class III contoured at σ=3.5. Extra density outside of the AAA+ ring domains are colored with the central density connecting to the pore in cyan, and the handle-like density in magenta. **b** Phenix-refined AAA1 and AAA2 domains fit into the cryo-EM map of ATAD2 Walker B mutant-H3/H4K5Q complex Class III. **c** Model of a single ATAD2 bromodomain (PDB ID: 3DAI) fit into the handle-like extra density. Bromodomain is rainbow-colored from N- to C-terminus.

SUPPLEMENTARY FIG 13

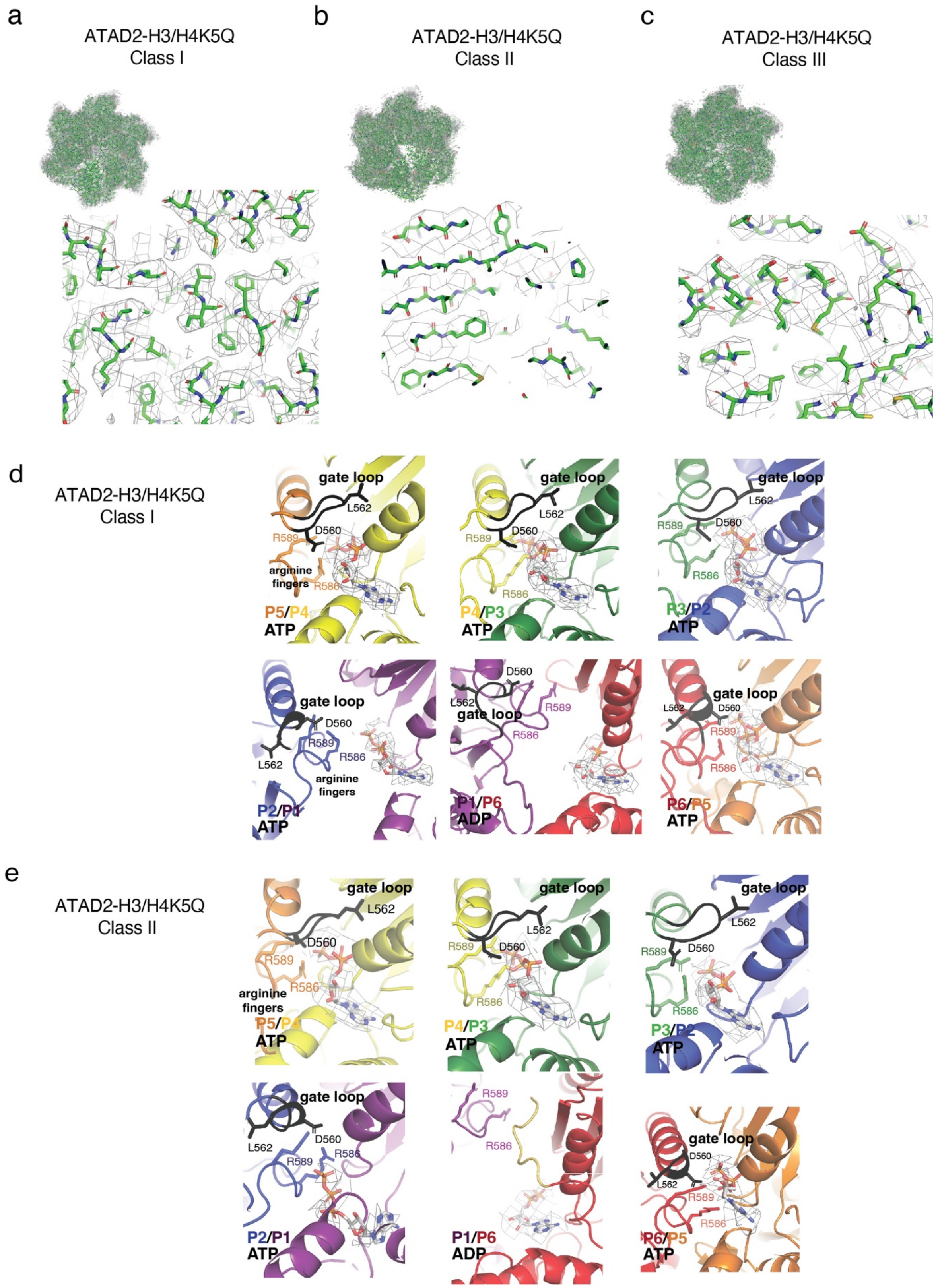

Supplementary fig 13. Density maps and nucleotide pockets of ATAD2 Walker B mutant - H3/H4K5Q complex structures

a-c Cryo-EM map and model fit of ATAD2 Walker B mutant-H3/H4K5Q complex Class I (**a**), Class II (**b**), and Class III (c). **d-e** Closeup view of nucleotide binding pockets of ATAD2 Walker B mutant-H3/H4K5Q complex Class I (**d**) and Class II (**e**) showing nucleotide identity, gate loop position, and arginine fingers. Density map for nucleotide (contoured at σ=4.5) is shown and arginine finger residues R586 and R589 are labeled. α3- β4 gate loops are labeled in black with side chains of conserved residues D560 and L562 shown.

SUPPLEMENTARY FIG 14

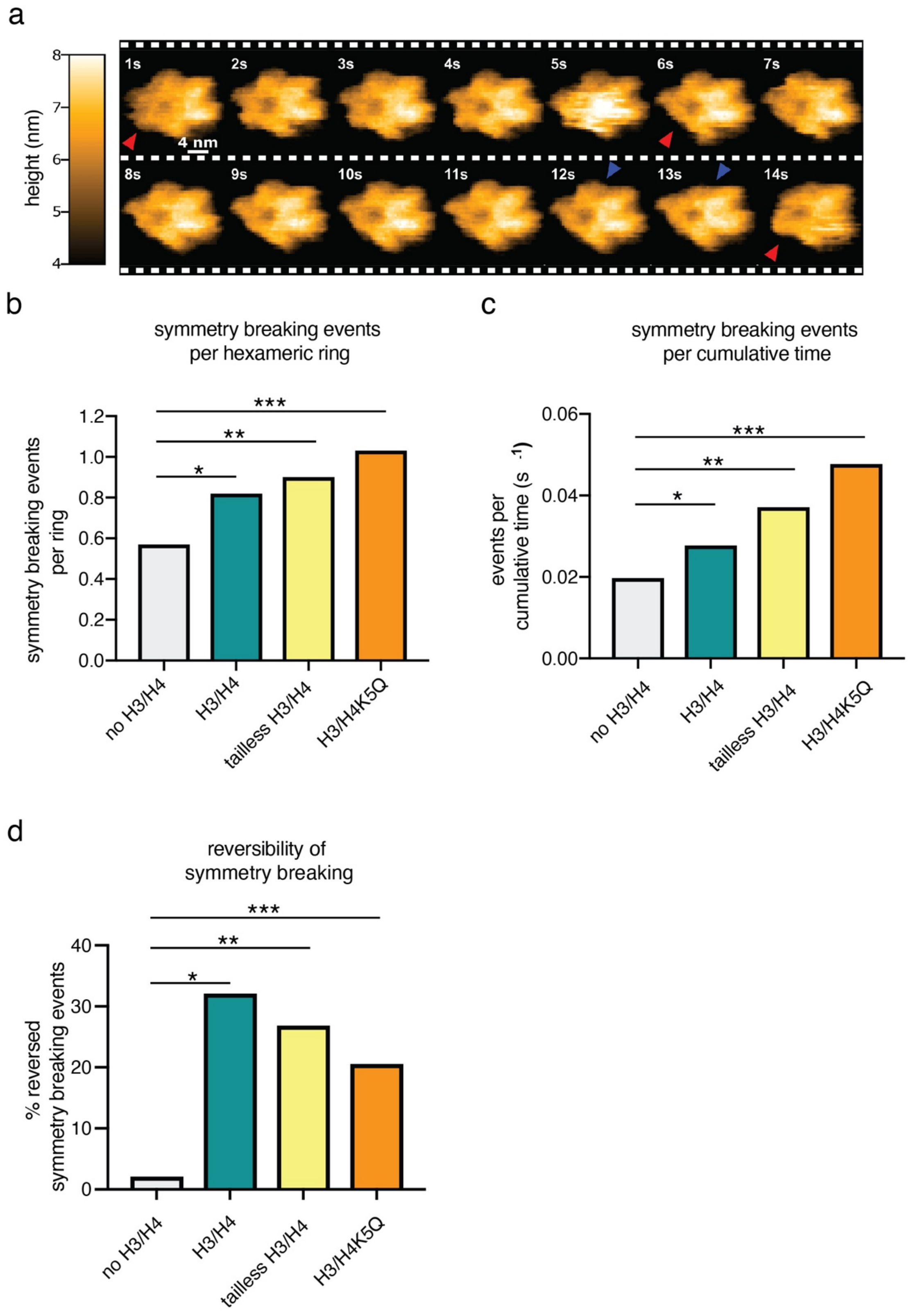

Supplementary fig 14. HS-AFM observation of symmetry breaking

a Selected frames from a representative HS-AFM movie of ATAD2 hexameric ring symmetry breaking taken at 2 Hz. Red arrowhead indicates a disappearing blade that reappears (reversible symmetry breaking event), and blue arrowhead indicates a disappearing blade that does not reappear during the span of the movie (irreversible symmetry breaking event). **b** Frequency of ATAD2 symmetry breaking as quantified by symmetry breaking events per ring. (For each group, number of rings observed (N) = 79, 32, 91, 112, for no H3/H4, H3/H4, tailless H3/H4, and H3/H4K5Q). P-values when comparing H3/H4, tailless H3/H4, and H3/H4K5Q to no H3/H4 are P(*)=0.069, P(**) < 0.01, P(***)= 0.001). **c** Frequency of ATAD2 symmetry breaking as quantified by symmetry breaking events per cumulative observation time. Cumulative observation time is 2300 s, 1100 s, 2200 s, 2300 s, respectively for no H3/H4, H3/H4, tailless H3/H4, and H3/H4K5Q. P-values when comparing H3/H4, tailless H3/H4, and H3/H4K5Q to no H3/H4 are P(*) =0.051, P(**) < 0.001, P(***) < 0.001. **d** Reversibility of ATAD symmetry breaking as defined by the re-appearance of a blade that has disappeared. P-values when comparing H3/H4, tailless H3/H4, and H3/H4K5Q to no H3/H4 are P(*)=0.085, P(**) < 0.01, P(***) =0.017

SUPPLEMENTARY METHODS

Gel filtration chromatography

Gel filtration was run on a Superose 6 increase 10/300 GL column (Cytiva Life Sciences) connected to an AKTA FPLC chromatography system. Molecular weight calibration of the column was performed by running thyroglobulin (670 kDa), ferritin (440 kDa), and aldolase (158kDa) from the gel filtration HMW Calibration Kit (Cytiva Life Sciences). For gel filtration of ATAD2, column was equilibrated with 1.5 column volumes of buffer (25 mM HEPES (pH 7.5), 250 mM NaCl, 5% glycerol, and 1 mM DTT) with appropriate nucleotide condition, and followed by injection with 500 µL of concentrated wild type or mutant ATAD2 protein.

Native PAGE

Native gel electrophoresis of was performed by loading 1-2 µg of protein sample mixed with 5% glycerol on a Native PAGE^{TM} 4-16% Bis-Tris minigel (Thermo Fisher Scientific and running for 2.5 h at 150 V at 4 °C. Gels were stained with Coomassie blue stain for 5 min, and destained for > 10 min with a 10% acetic acid, 40 % methanol destaining solution.

Negative Stain EM

Carbon-coated Pelco GC400 mesh copper grids (Ted Pella, Inc.) were glow discharged with a PELCO easiGlow glow discharge cleaning system (Ted Pella, Inc.) with a current of -30 mA, a hold time of 10 s, and a glow time of 20 s. Subsequently, 3 µL of ATAD2 protein diluted to 0.01-0.05 mg/mL was applied to the glow discharged grid followed by incubation for 1 min. After incubation, excess protein solution was removed by blotting on filter paper. The grids were then washed 2 times by dipping in 10 µL water and blotting on filter paper, and stained by

dipping in 10 μL of 1.5% uranyl acetate solution for 1 min. Excess stain was removed by blotting and grids were dried for 1 min and stored in a dessicator until observation. EM grids were observed on a 200kV Tecnai F20 transmission electron microscope (FEI) at 29,000x or 38,000 x with a CCD detector (Gatan) at the KAIST Analysis Center for Research Advancement (KARA). 2D class averages of particles from negative stain EM were obtained in Relion 4.0 by initially picking ~5,000 particles per sample, and performing iterative 2D classification and selection of aligned classes.

Crosslinking mass spectrometry (XL-MS)

ATAD2-histone H3/H4 complexes were prepared by the same method as for cryo-EM samples, with the exception that 1 mM of DSS H12/D12 (Creative Molecules) was used instead of 1 mM DSS. Crosslinked ATAD2-histone H3/H4 complexes were purified on a 10-30% sucrose gradient as with cryo-EM samples and buffer-exchanged to 25 mM HEPES (pH 7.5), 250 mM NaCl, 5% glycerol, 1 mM DTT, and 1 mM Mg-ATP. Denaturation, blocking, and digestion of proteins were performed essentially as in[1]. Digested peptides were separated by passage over a Superdex peptide 3.2/30 column, and analyzed on an Orbitrap mass spectrometer (Thermo Fisher Scientific) at the Taplin Mass Spectrometry facility at Harvard Medical School. Data analysis was performed using xQuest[2] and a sequence database containing ATAD2, histone H3/H4 sequences. xQuest search results were filtered according to the following criteria: mass error < 4 ppm, minimum peptide length = 6 residues, delta score < 0.9% TIC ⩾ 0.1, minimum number of bond cleavages per peptides = 4, and an xQuest LD score cutoff of 25 was selected, corresponding to a false discovery rate of < 2%. Final crosslinks were filtered and visualized with xiView[3].

High-speed atomic force microscopy measurements

HS-AFM images were recorded at frequency of 2 Hz with a laboratory-made instrument exclusively in tapping mode.[4] The cantilevers were oscillated around the resonance frequency with a free amplitude between 1 and 2 nm. The setpoint for the feedback control was 80% of the free oscillation amplitude. A mica disc with 1.5 mm diameter was used a substrate for ATAD2 deposition. Directly before ATAD2 adsorption, the topmost mica layers were cleaved and a solution of 0.1% (3-Aminopropyl) triethoxysilane (APTES) diluted by milliQ water was incubated for 3 min to modify the surface to a positively charged state. Following the flushing of APTES with 20 µL milliQ water, 20 nM ATAD2 in observation buffer (25 mM HEPES (pH 7.5), 150 mM NaCl, 5 % glycerol, and 1 mM DTT) was deposited and incubated for 5 min to facilitate adsorption. For experiments with H3/H4, 46 nM H3/H4 was mixed with 20 nM ATAD2 and incubated for 10 min before adsorption to the aminosilane-coated mica substrate. ATP was added to the observation buffer before HS-AFM observation to a final concentration of 2 mM.
The cantilevers used for HS-AFM measurements were 9 µm long, 2 µm wide, had a spring constant between of 0.1 and 0.3 N/m, and a resonance frequency in buffer around 700 kHz. Carbon tips were grown by electron deposition at the very end of the cantilever and consecutively plasma etched to achieve an apex radius between 2 and 5 nm.

HS-AFM image analysis

Individual ATAD2 rings were tracked to remove drift and cropped from the raw images to a size of 30 nm × 30 nm to include only a single ring. This single ring was then low-pass filtered to cut off lateral frequencies below 1 nm. The filtered rings were viewed frame by frame until a symmetry breaking event occurred or observation was stopped. During this period, the

number of symmetry-breaking events was counted, excluding full disassembly or detachment of a hexameric ring. The number of reversible symmetry breaking events (events where individual blades disappeared and reappeared) were also counted. In addition, the frame count until the end of the observation was recorded for each ring. To quantify the symmetry breaking frequency for each condition, symmetry breaking events per hexameric ring observed (total number of symmetry breaking events/total number or hexameric rings), symmetry breaking events per time (total number of symmetry breaking events/total observation time), and reversibility (total number of reversible symmetry breaking events/total number of symmetry breaking events) was quantified for each experimental condition, To test for statistical significance, different experimental conditions were subject to a Welch test.

Preparation of tailless H3/H4

To obtain H3- and H4-tail truncated H3/H4, purified wild type H3/H4 was trypsinized as in Cho et al.[1] Briefly, 50 µL of concentrated H3/H4 at 230 µM was mixed with of 15 µL of immobilized TPCK trypsin (ThermoFisher Scientific), and incubated at 4°C for 30 min. Reactions were quenched by removal of immobilized trypsin by Millipore Ultrafree-MC centrifugal filters (Merck). Trypsinized H3/H4 was examined by SDS-PAGE showing a size decrease to the size of the histone body, and binding to ATAD2 was confirmed by comparing titration of ATAD2 on a native gel with wild type and tailless H3/H4.

SUPPLEMENTARY REFERENCES

1. Cho, C. et al. Structural basis of nucleosome assembly by the Abo1 AAA+ ATPase histone chaperone. *Nat Commun* **10**, 5764 (2019).
2. Leitner, A., Walzthoeni, T. & Aebersold, R. Lysine-specific chemical cross-linking of protein complexes and identification of cross-linking sites using LC-MS/MS and the xQuest/xProphet software pipeline. *Nat Protoc* **9**, 120-37 (2014).
3. Graham, M., Combe, C., Kolbowski, L. & Rappsilber, J. xiView: A common platform for the downstream analysis of Crosslinking Mass Spectrometry data. *bioRxiv*, 561829 (2019).
4. Ando, T. et al. High-speed AFM and nano-visualization of biomolecular processes. *Pflugers Arch* **456**, 211-25 (2008).
