# Supplementary material for: Structure of the human ATAD2 AAA+ histone chaperone reveals mechanism of regulation and inter-subunit communication
Source: Commun Biol. 2023 Sep 28;6:993. doi: 10.1038/s42003-023-05373-1 (PMC10539301; doi:10.1038/s42003-023-05373-1)
Supplement: Supplementary file 3 — Description of Additional Supplementary Files [file 42003_2023_5373_MOESM3_ESM.pdf]

Description of Additional Supplementary Files

File name: Supplementary Movie 1
Description: Morph of ATAD2 monomeric subunit structure.

File name: Supplementary Movie 2
Description: Morph of ATAD2 with ATAD2-Histone H3/H4 complex class I structure.

File name: Supplementary Movie 3
Description: Morph of ATAD2 with ATAD2-Histone H3/H4 complex class II structure.

File name: Supplementary Movie 4
Description: Morph of ATAD2-Histone H4/H4 complex I, III, and II structure.

File name: Supplementary Data file 1
Description: Comparison of peptide crosslinks in ATAD2 vs. ATAD2-H3/H4.
